# Tailoring immunisation programmes in a time of SARS-CoV-2: What can be learnt by comparing the findings of childhood and COVID-19 vaccine evaluation studies in an underserved population?

**DOI:** 10.1016/j.puhip.2022.100287

**Published:** 2022-07-02

**Authors:** Ben Kasstan, Louise Letley, Sandra Mounier-Jack, Nicole Klynman, Katherine M. Gaskell, Rosalind M. Eggo, Michael Marks, Tracey Chantler

**Affiliations:** a Centre for Health, Law & Society, University of Bristol, Bristol, BS8 1RJ, United Kingdom; b Department of Sociology & Anthropology, Hebrew University of Jerusalem, Har HaTzofim, Jerusalem, 91905, Israel; c Immunisation and Countermeasures, National Infection Service, UK Health Security Agency, London, United Kingdom; d The Vaccine Centre, Department of Global Health and Development, London School of Hygiene & Tropical Medicine, 15-17 Tavistock Place, London, WC1H 9SH, United Kingdom; e City and Hackney Public Health Team, Hackney Council, 1 Hillman Street, London, E8 1DY, United Kingdom; f Clinical Research Department, Faculty of Infectious and Tropical Diseases, London School of Hygiene & Tropical Medicine, Keppel Street, London, WC1E 7HT, United Kingdom; g Centre for Mathematical Modelling of Infectious Diseases, London School of Hygiene & Tropical Medicine, Keppel Street, London, WC1E 7HT, United Kingdom; h Hospital for Tropical Diseases, University College London Hospital NHS Foundation Trust, London, WC1E 6JB, United Kingdom

**Keywords:** Covid-19, Commissioning, Qualitative research, Tailoring Immunization Programme, United Kingdom

## Abstract

**Objectives:**

A WHO Tailoring Immunization Programmes (TIP) evaluation was conducted in 2014-16 to investigate suboptimal childhood vaccination coverage in the north London Orthodox Jewish community. In 2021-22 a qualitative evaluation of the COVID-19 vaccine programme (CVP) was conducted in the same setting. This paper examines whether the issues identified by the TIP affected the CVP and what differences emerged between these two vaccine programme evaluations.

**Study design:**

Qualitative study.

**Methods:**

The CVP evaluation involved conducting 28 semi-structured interviews with public health professionals, Orthodox Jewish welfare and religious representatives, and household members in February-May 2021. The key considerations arising from the thematic analysis of this data was then compared systematically with the overarching findings from the TIP study.

**Results:**

The issues identified in the TIP study diverged and converged with results from the CVP evaluation: i) participants did not express concerns of unmet CVP information needs; ii) the social value of COVID-19 vaccines was influenced by international travel requirements; iii) in contrast to commissioning constraints noted to have limited flexible delivery of childhood immunisations in the TIP evaluation, the CVP was characterised by a flexible commissioning and delivery model. This model was facilitated by significant government investment as part of the COVID-19 pandemic response.

**Conclusions:**

The comparative analysis indicates that flexible vaccine commissioning and fit for purpose public health investment can influence how documented knowledge is translated into action. Implications are raised for how routine vaccination services are equipped to serve the needs of minority populations with historically suboptimal coverage levels.

## 1 Introduction

One of the first WHO Tailoring Immunization Programmes (TIP) studies was conducted in 2014-16 with an Orthodox Jewish minority in London due to suboptimal childhood vaccination coverage levels leading to persistent measles outbreaks [1,2]. This minority population was subsequently found to have extremely high SARS-CoV-2 seroprevalence rates in 2020 [3], and COVID-19 vaccination rates have been lower than the national average in north London [4]. The aim of this paper is to i) examine the extent to which the same issues arose in the TIP and implementation of the COVID-19 vaccination programme (CVP) in London; ii) what evidence there is to suggest that TIP findings had improved vaccination delivery strategies, iii) and what accounts for any differences between the TIP and CVP evaluation findings.

### 1.1 TIP methodology and 2014-16 study

**Fig. 1:**
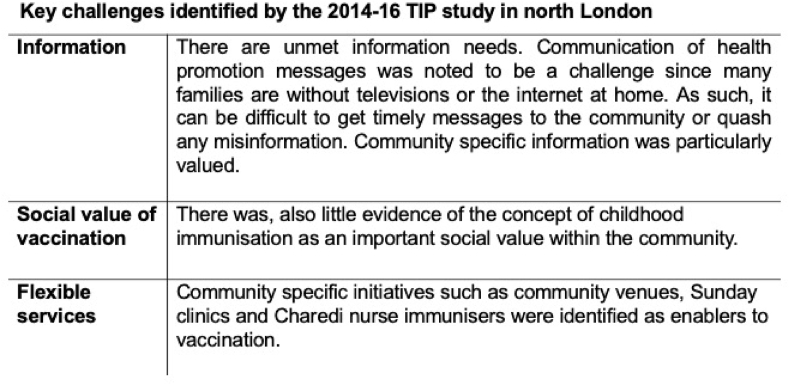
Key challenges identified by the 2014-16 TIP study in north London.

The TIP approach has been refined based on the study conducted in north London in 2014–16. The TIP methodology was developed by the WHO Regional Office for Europe to ‘integrate people-centred research and behavioral insights into immunization programme planning and policy.’ [5] TIP operates on three logics: i) identifying populations or geographic areas with suboptimal vaccination coverage, ii) determining barriers and drivers to vaccination, and iii) using these situated (context-specific) insights to design evidenced-based interventions for high and equitable vaccination uptake [4]. Community engagement and ‘consideration of the wide range of behavioural determinants affecting vaccine uptake’ form a key conceptual framework in projects applying a TIP approach [6]. Three key challenges [2] were identified by the 2014-16 TIP study conducted in an Orthodox (Haredi) Jewish minority of London (Fig. 1).

The key challenges identified by the 2014-16 TIP study are interdependent. Attempts to address information needs, for example, by producing Yiddish or Hebrew translations, or paying for advertisements in local Jewish press, were susceptible to short term funding arrangements [1]. Hence, issues in information needs and attempts to promote childhood vaccination as a norm are profoundly linked to commissioning constraints, and the framework within which flexible services can operate and be responsive to situated needs and expectations.

### 1.2 CVP

In England, the CVP was launched in December 2020 with priority initially based on age and clinical vulnerability. The CVP was delivered by a range of NHS providers, including general practices networked through primary care networks (PCNs), and hospital trusts, some of which were commissioned to operate mass vaccination hubs and in some instances pop-up clinics. The CVP received unprecedented funding, some directed to addressing vaccine inequalities. Local authorities were concerned that ethnic and religious minority groups, including Orthodox Jews, would be less likely to accept COVID-19 vaccines, prompting discussion on how to offer tailored communications and services [7]. The CVP was co-delivered in London with a Haredi volunteer emergency medical service, *‘Hatzolah*,' via a series of dedicated vaccination sessions [8]. Local public health teams and *Hatzolah* maintained a clear division of responsibility, with latter being responsible for circulating information about the CVP, booking appointments, and administering vaccines [8].

### 1.3 Comparing the 2014-16 TIP study and CVP

In this paper we examine whether the key issues identified by the TIP also affected the CVP and what differences emerged between these two programmes. We did this by drawing on data from a twelve-month qualitative study examining the delivery of the CVP in London [7].

## 2 Methods

**Fig. 2:**
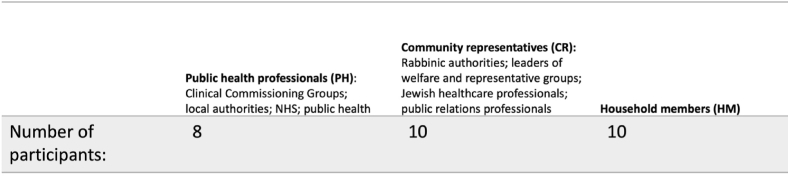
Research clusters and numbers of participants interviewed.

This qualitative research study consisted of 28 semi-structured interviews examining responses to the UK CVP in a Haredi neighbourhood in London (Fig. 2), between February and May 2021.

### 2.1 Participant recruitment and particulars

Participants were recruited from on-going research collaborations with Public Health England (now UK Health Security Agency), local authorities (LA) in London, and with Haredi families, and via snowball sampling. The named authors include social scientists with extensive experience of service evaluation and engagement with Haredi families, and public health professionals (UKHSA and LA) − which enabled professional networks to be maximised for snowball sampling.

We evaluated the co-delivery of the CVP in London based on the perspective of public health (PH) providers, community representatives (CR), and household members (HM) (Fig. 2). A range of PH professionals were interviewed (Fig. 2) because vaccine programme responsibilities in England are delegated across National Health Service (NHS) organisations, Clinical Commissioning Groups (CCGs), Health Protection Teams, General Practice, and Directors of public health. Community representatives consisted of rabbinic authorities, leaders of Haredi welfare organisations, Jewish healthcare professionals, and public relations representatives. Household members ranged in age, gender, educational and professional background, and the Haredi movement to which they affiliate. The particulars of their affiliations have been removed for anonymity.

### 2.2 Analysis

Interviews lasted between 30 and 90 mins and were recorded with participant consent. Analysis of the interview data was inductive and thematic, whereby theoretical insights emerge from prolonged engagement with the data rather than being pre-conceived [9,10]. Interviews conducted with PH professionals raised issues surrounding the application of findings from the 2014-16 TIP study in London, which emerged as a core area of analysis. The data was analysed by BK and TC, who initially coded the same 6 transcripts as a test of reliability.

### 2.3 Research ethics

Ethical approval to conduct this study was provided by the London School of Hygiene & Tropical Medicine (reference: 22532).

## 3 Findings

Results illustrate that the challenges documented in the TIP study diverged and converged with the CVP, and can be summarised as: i) participants did not express concerns of unmet CVP information needs; ii) the social value of COVID-19 vaccines was influenced by international travel requirements; iii) the CVP was characterised by a flexible commissioning and delivery model, which differed to the barriers affecting childhood vaccinations.

### 3.1 Information

Information needs around the CVP were met through a series of collaborations between the local authority, public health professionals and community representatives, and hence differed from the issues of unmet information needs raised by the TIP study. Firstly, Jewish newspapers with an exclusive Orthodox readership sought to circulate central government messaging around the CVP and the local authority paid for announcements to be printed in neighbourhood-run circulars. Secondly, an Orthodox Jewish rapid response service (*Hatzolah*) had co-delivered [7] COVID-19 vaccination sessions as part of a Primary Care Network-run local COVID vaccination centre, and produced advertisements, both in print and digitally. Thirdly, an example of the population-specific information channels that were initiated to promote the CVP included a telephone helpline, which was launched at the beginning of the pandemic with local authority funding and was later used by HMs to ask questions about the CVP:

‘We were getting calls to the helpline. The council took all that, put it together and got a doctor in the community to do a response, like commonly asked questions about the vaccine. So, will the vaccine work on the new coronavirus variant? Is there reliable evidence of long-term impact of the vaccine? Will the vaccine make you infertile? Are there any side effects?’ (CR3)

### 3.2 Social value

The TIP study raised questions as to what social norms or values exist around childhood vaccinations, but based on analysis of the CVP it was clear that COVID-19 vaccines were valued for a range of reasons. At the time of study, media attention had raised speculation about the possibility of ‘vaccine passes’ for international travel. Against this backdrop, household members and community representatives described how COVID-19 vaccines were valued for travel, especially to be reunited with family in Israel, but also as part of pandemic recovery in the UK and getting back to “normal” – even if this meant accepting vaccines reluctantly:

‘Getting the vaccine is probably going to help you get back to normal as soon as possible. My wife went out and made sure she got the vaccine because her parents and grandparents live in Israel. So, for her, it was very important. There was this sort of wink of “you will be able to travel if you’re vaccinated,” so that’s the biggest enticement to get it. So, you do it, but you do it reluctantly.’ (HM7)

### 3.3 Service flexibility and funding

Public health professionals cited knowledge of the key TIP findings, notably around the need for flexible and accessible childhood vaccination services due to larger family sizes. However, participants suggested that the ability to address the issues of flexible and accessible services identified by TIP were hampered by financial cuts that affected the ability of local delivery strategies to operationalise services that would resolve convenience issues for this minority. As recommended by the TIP study, public health professionals applied a flexible approach in the implementation of the CVP and were able to collaborate with community stakeholders to enable the CVP to be delivered locally.

‘It’s why the vaccination events we’ve done on Saturday nights after Sabbath have been so successful. People want stuff on their doorsteps. We’ve not had much of an issue with take-up of the vaccine in that community, because we’re still working through the older cohorts.’ (PH4)

A key difference between childhood (at the time of the TIP study) and COVID-19 vaccination programmes were the commissioning strategies. The absence of a well-resourced and flexible strategy was cited as a barrier to reversing unmet childhood vaccination needs of this minority, whereas the CVP was able to tailor implementation due to its funding model:

‘It’s not one [issue], it’s multi-dimensional and you need to wrap around all of these things. I think there were issues with – how do you say it – what didn’t happen [following TIP]. Nothing was put in place and not for want of trying but the money doesn’t sit with the local authority at that point for imms [immunisations]. The money doesn’t sit with the community.’ (PH2)

## 4 Discussion

Results indicate evidence of progress in the delivery of vaccination programmes in London since the issues identified in the TIP study [1,2]. The TIP study identified unmet information needs regarding routine immunisation. Findings from the CVP study demonstrated that public health and Orthodox Jewish stakeholders collaborated to promote information about the CVP using both print and digital means – and HMs received these messages. As noted previously, misinformation [8] did circulate in north London, but this was consistent with issues observed among the broader UK population [11]. Hence, a lack of CVP information does not appear to be the problem in the way it was noted to be for childhood vaccinations by the TIP study [2]. The prolific circulation of information pertaining to the CVP in 2020–21, however, did not always mean that COVID-19 vaccines would be accepted by Haredi HMs without reservation. The CVP was newly developed and rapidly implemented from December 2020, just a few months prior to the time of study (February to May 2021), which is, in reality, a modest period of time to promote confidence and expect public buy-in. The sustainable allocation of public funds to develop situated and population-specific interventions, such as telephone lines and printed and digital advertisements, were valued for offering accessible and targeted information via multiple channels. The TIP study and our evaluation of the CVP highlight the importance of collaboration between public health, minority stakeholders and household members in the development and dissemination of vaccination information.

Questions remain around interpretations of social values or norms with regards to vaccination. We argue that there is a need to define these terms and consistently apply indicators in order to measure vaccination values [14,15] or norms across populations. This is important to avoid stigmatising minority populations and to generate appropriate interventions. Understanding social values attributed to vaccination in this population is important for vaccine delivery strategies beyond the UK context, as major measles outbreaks emerged in Haredi neighbourhoods in Israel and the US in 2018–19 [[16], [17], [18], [19]]. Extremely high COVID-19 seroprevalence rates have since been recorded in Haredi populations in the US [20], and COVID-19 vaccination uptake is lower among Haredi neighbourhoods in Israel [21].

The PH professionals we interviewed in 2021 noted that the 2014-16 TIP recommendations had informed the way they liaised with community groups to tailor the delivery of childhood vaccination programmes and improve vaccine communication. The delivery of flexible services (e.g. clinics in children’s centres) had however proved difficult due to funding limitations. These immunisation programme funding limitations are in part a legacy of the large-scale reorganisation of the NHS in 2013. This reorganisation resulted in significant fragmentation in the way that the immunisation programme was commissioned and delivered. The most significant change was the redeployment of experienced immunisation staff across new and revised organisations: NHS England Screening and Immunisation Teams (responsible for commissioning immunisation service providers) and Local Authority Public Health teams (responsible for assuring that their populations are protected against infectious disease) [12]. Notably our 2021 PH interlocuters described that this infrastructure had resulted in a less coordinated approach to the delivery of vaccine programmes in local areas and made it more difficult for them to fund innovative outreach to underserved populations. The CVP offered a departure from this, due to never-before-seen levels of government investment [13] and flexible methods of delivery. The improvements in addressing delivery needs that were observed in the CVP raise implications for the delivery of childhood vaccines, especially given the rise in measles cases in this area and in recent years and the country more broadly.

We have argued elsewhere for a ‘localised’ model of vaccination delivery, where services are not only tailored in a ‘convenient and culturally appropriate manner, but localised and co-delivered with welfare groups that are valued, trusted and managed within minority settings.’ [8] Based on the comparison in this paper, we suggest that a ‘localisation’ model might help to translate the situated (context-specific) recommendations that arose from the TIP study. The design of localised immunisations services (childhood and adult) will of course require close collaboration with those currently responsible (e.g. GPs, CCGs, local authorities) for delivery to identify gaps (e.g. suboptimal uptake of specific vaccines) in services provision and consider how localised approaches could help address inequalities.

Previous studies examining how large-scale restructuring of healthcare systems affect vaccination programmes have noted the importance of maintaining ‘institutional memory’ for effective public health delivery strategies [12]. How institutional knowledge is documented and implemented is a core issue of evaluation, and our results indicate the need for commissioning of services to enable research findings to be actioned quickly. A sustainable strategy is required to commit funds and invest in flexible vaccine commissioning — or else the public health ability *to serve* populations is compromised.

### 4.1 Strengths and limitations

A strength of our approach is examining how challenges documented in a past TIP study differed to the CVP, which raises an opportunity to learn from the production of knowledge and to support attempts to maintain sustained protection against outbreaks of infectious disease in this minority. The TIP and our study of the CVP took place at different times, and also differ in that the former is concerned with childhood vaccinations and the CVP has a broader remit of people aged 12 and above. While we maintain that a comparative approach is helpful, we should be cautious about drawing inferences given the difference in the scales of the vaccination programmes, their different commissioning arrangements, and the context of urgency [22] produced by the COVID-19 pandemic. Further evaluation of best practice is required to enhance COVID-19 coverage levels, which remain suboptimal among areas that are home to Haredi populations [4,21].

## 5 Conclusion

Key issues identified in the TIP study led to recommendations that can strengthen vaccination programmes. The localised approach adopted in the CVP reflects some of the key TIP recommendations on implementing flexible servicing. Overall, our findings suggest re-envisioning vaccine commissioning to be open to plural models of implementation, with support of public health and primary care. Using ‘localised’ [8] approaches model may be an integral way of implementing knowledge produced by evaluations, such as the 2014-16 TIP study. Localised approaches may also help to nurture long-term confidence in vaccination among populations with suboptimal levels of vaccine coverage leading to persistent outbreaks of preventable disease. Hence, our study raises implications for how vaccination services are commissioned and funded *to serve* the needs of minority groups with historically suboptimal coverage levels.

## Funding

This work was jointly funded by 10.13039/100014013UKRI and 10.13039/501100000272NIHR [COV0335; MR/V027956/1], a donation from the 10.13039/100009660LSHTM Alumni COVID-19 response fund, HDR 10.13039/100007472UK, the 10.13039/501100000265MRC and the 10.13039/100010269Wellcome Trust (210830/Z/18/Z). This research was supported by the 10.13039/501100000272National Institute for Health Research Health Protection Research Unit (NIHR HPRU) in Immunisation.

Tracey Chantler, Ben Kasstan, Louise Letley, and Sandra Mounier-Jack are affiliated to the National Institute for Health Research Health Protection Research Unit (NIHR HPRU) in Vaccines and Immunisation (NIHR200929) at London School of Hygiene and Tropical Medicine in partnership with UK Health Security Agency (UKHSA). Tracey Chantler, Ben Kasstan, and Sandra Mounier-Jack are based at London School of Hygiene & Tropical Medicine and Louise Letley is based at UKHSA. The views expressed are those of the author(s) and not necessarily those of the NHS, the NIHR, the Department of Health or UKHSA.

## Author contributions

TC, MM, RE and BK conceived of the study. BK and TC planned and conducted the qualitative data collection and led the data analysis. KG, LL, NK, SMJ contributed to the design of the study. All authors reviewed the analysis and contributed to writing the manuscript.

## Ethics

Ethical approval to conduct this study was granted by The 10.13039/100009660London School of Hygiene and Tropical Medicine Research Ethics Committee (Ref: 22532). Due to confidentiality agreements with participants as part of the consent process the interview data reported in this manuscript cannot be shared and is not available for secondary analysis.

## Declarations of compeying interest

BK, SMJ, KG, RE, MM, NK, TC have no declaration of interests to declare.
